# Long term evaluation of patient satisfaction and quality of life in patients undergone successfuL Nuss procedure

**DOI:** 10.1186/s13019-025-03660-y

**Published:** 2025-11-05

**Authors:** Sule Karadayi, Ayse Ugurum Yucemen, Ozgur Katrancioglu

**Affiliations:** 1 Department of Thoracic Surgery, Cumhuriyet University School of Medicine, Sivas, Turkey; 2 Department of Thoracic Surgery, Sivas Numune Hospital, Sivas, Turkey

**Keywords:** Pectus excavatum, Patient satisfaction, Minimally invasive surgery, Single-Step questionnaire

## Abstract

**Background:**

Pectus excavatum represents the most common chest wall deformity, and surgical correction via the Nuss procedure has gained widespread adoption. Long-term patient satisfaction and quality of life remain inadequately characterized, particularly with respect to age-dependent outcomes and factors influencing satisfaction. To evaluate long-term patient satisfaction and quality of life in pediatric, adolescent, and adult patients who underwent successful Nuss procedures and to identify factors predictive of sustained improvement.

**Methods:**

We conducted a retrospective analysis of 40 patients who underwent the Nuss procedure between 2010 and 2020. Patient satisfaction and quality of life were assessed via the single-step questionnaire (SSQ). Patients were stratified by age groups: children (≤ 12 years), adolescents (13–17 years), and adults (≥ 18 years). Statistical analyses included comparisons between age groups and genders, correlation analyses, and multiple regression to identify predictors of satisfaction.

**Results:**

Patients demonstrated favorable long-term outcomes. Pediatric patients presented significantly higher scores in the physical functioning, pain/discomfort, and total SSQ domains than adolescents and adults did. Age at operation was negatively correlated with satisfaction outcomes. Preoperative chest pain was associated with lower satisfaction. Patients with primarily cosmetic indications reported greater self-esteem improvement and total satisfaction. Multiple regression analysis revealed that advanced age at operation, preoperative chest pain, and postoperative chronic pain were significant negative predictors of satisfaction.

**Conclusions:**

The Nuss procedure provides durable patient satisfaction across multiple quality-of-life domains, with age-dependent variations. Earlier intervention, when clinically appropriate, may optimize long-term outcomes. Effective management of both pre- and postoperative pain is crucial for maximizing patient satisfaction.

## Introduction

Pectus excavatum (PE) is the most common congenital chest wall deformity, affecting approximately 1 in 300–400 live births. While often considered cosmetic, it may lead to symptoms such as reduced exercise tolerance and cardiopulmonary compromise in severe cases [1–3]. The minimally invasive repair of PE (MIRPE), also known as the Nuss procedure, has become the standard surgical treatment. Although short-term outcomes are well established, long-term patient-reported outcomes remain relatively underexplored [4–7].

In recent years, patient-reported outcome measures (PROMs) have gained importance in assessing quality of life following surgical correction, particularly for PE, where cosmetic and psychological factors are primary motivations for intervention. The Single-Step Questionnaire (SSQ) is a validated, concise tool for evaluating postoperative satisfaction in this population. However, there is a lack of data on diverse age groups and long-term follow-up beyond bar removal [8–10].

This study aims to evaluate long-term patient satisfaction and quality of life in a cohort of patients who underwent the Nuss procedure at our institution. Using the SSQ, we assessed outcomes at least five years postoperatively, stratified by age and gender. The findings are intended to contribute observational data regarding postoperative satisfaction from a single-center experience.

## Materials and methods

After institutional review board approval (approval number: 2023-12/31), we conducted a retrospective analysis of patients who underwent pectus excavatum repair via the Nuss procedure at our institution between January 2010 and December 2020. Eligible patients included those who had completed at least five years of follow-up after their surgical procedure. Patients with a history of connective tissue disorders, recurrent pectus excavatum, or those who underwent combined cardiothoracic procedures were excluded from the study.

Demographic information, operative details, and clinical outcomes were extracted from medical records. Preoperative assessment included physical examination, cardiac evaluation (electrocardiogram and echocardiography), pulmonary function tests, and computed tomography scanning. The severity of pectus excavatum deformity was quantified via the Haller index, with a value greater than 3.5 considered indicative of severe deformity.

The primary indication for surgical intervention was cosmetic concerns in the majority of cases, although some patients also presented with cardiopulmonary symptoms or body image issues. The study population comprised pediatric, adolescent, and adult patients, with a small proportion of female participants. Additionally, a subset of patients elected to retain their pectus bars beyond the standard removal timeframe or indefinitely.

### Surgical technique

All patients underwent a conventional video-assisted Nuss procedure under general anesthesia. A thoracoscopic approach was utilized to ensure safe passage of the pectus bar. If a single metal bar did not satisfactorily correct the deformity, a second bar was placed superiorly. Each bar was stabilized with at least one lateral stabilizer to prevent displacement.

Postoperatively, patients received multimodal pain management, including epidural analgesia when appropriate, which was maintained for 3–5 days. Patients were carefully instructed regarding postoperative precautions, including sleeping in the supine position and avoiding sudden twisting or bending movements of the thorax. Physical activities were restricted during the first 4‒6 weeks after surgery, with a gradual return to normal activities thereafter. Bar removal was typically performed 2–4 years after the initial surgery.

### Long-term evaluation and quality of life assessment

For the assessment of long-term patient satisfaction and quality of life, we utilized the single-step questionnaire (SSQ), which was introduced by Krasopoulos et al. and validated for use in patients with pectus excavatum [6]. The questionnaire comprises 16 items measuring various domains, including general health perception, physical functioning, social belonging, chest pain/discomfort, and overall satisfaction (Table 1).

**Table 1:** Single-Step questionnaire items and scoring system

Question	Domain	Scoring
1. Health in general after the operation	Physical functioning	Much better now: 5, Somewhat better: 4, About the same: 3, Somewhat worse now: 2, Much worse now: 1
2. Postoperative exercise capacity	Physical functioning	Much better now: 5, Somewhat better: 4, About the same: 3, Somewhat worse now: 2, Much worse now: 1
3. Impact of chest deformity on social engagement before surgery	Social functioning	Extremely: 5, Quite a bit: 4, Moderately: 3, Slightly: 2, Not at all: 1
4. Extent that chest appearance interferes with postoperative social activity	Social functioning	Not at all: 5, Slightly: 4, Moderately: 3, Quite a bit: 2, Extremely: 1
7. Impact operation had on social life	Social functioning	Major improvement: 5, Improved: 4, No change: 3, Worse now: 2, A lot worse now: 1
5. Satisfaction with the overall postoperative appearance	Cosmetic satisfaction	Extremely satisfied: 5, Very satisfied: 4, Satisfied: 3, Dissatisfied: 2, Very dissatisfied: 1
6. Bothered by the surgical scars	Cosmetic satisfaction	Not at all: 5, Very slightly: 4, Slightly: 3, A little bit: 2, A lot: 1
15. Chest looks different	Cosmetic outcome	Major improvement: 5, Improved: 4, No change: 3, Worse now: 2, A lot worse now: 1
8. Preoperative self-esteem	Psychological functioning	Score: 1–10
9. Postoperative self-esteem	Psychological functioning	Score: 1–10
10. Pain during hospital stay	Pain/discomfort	None: 5, Very mild: 4, Mild: 3, Moderate: 2, Severe: 2, Very severe: 1
11. Pain interfering with day-to-day activity now	Pain/discomfort	Not at all: 5, Very slightly: 4, Slightly: 3, A little bit: 2, A lot: 1
12. Pain now	Pain/discomfort	No: 5, Occasionally: 4, Mild—no painkillers: 3, Mild—painkillers: 2, A lot: 1
13. Conscious about the metallic bar	Physical awareness	Not at all: 5, Slightly: 4, Moderately: 3, Quite a bit: 2, Extremely: 1
14. Overall satisfaction with the final result	Overall satisfaction	Extremely satisfied: 5, Very satisfied: 4, Satisfied: 3, Dissatisfied: 2, Very dissatisfied: 1
16. Going back, would you have the operation again	Decision satisfaction	Yes: 10, Unsure: 5, No: 0

Patients were contacted by telephone or electronic communication for study enrollment, and upon receiving informed consent, questionnaires were delivered via email. A research assistant who was not part of the surgical team and was blinded to patient characteristics collected the completed questionnaires. For patients with retained pectus bars, the questionnaire was administered at least five years after the initial procedure.

The total score from the SSQ was calculated by summing the scores of all the items, with a maximum possible score of 84. For the self-esteem assessment (questions 8 and 9), the difference between the postoperative and preoperative scores was calculated and used as a contributor to the total score. A total SSQ score greater than 52 was considered indicative of a satisfactory long-term outcome. While the original SSQ validation by Krasopoulos et al. defined a satisfactory result as a score above 41, we applied a more conservative threshold. This decision was based on two considerations: First, the score distribution observed in our own patient cohort; and Second, the findings reported by Zuidema et al. who presented mean total SSQ scores ranging from 56.9 to 59.9 in a longitudinal cohort across multiple postoperative intervals, suggesting that higher scores are reflective of sustained satisfaction over time [11]. Although no fixed cutoff was defined in their study, their results support the use of > 52 as a meaningful threshold for high satisfaction. Furthermore, as recommended in contemporary scale development literature, thresholds in adapted or applied measurement tools can be contextually determined based on the sample characteristics and distributional behavior of the instrument within the studied population [12].

### Statistical analysis

Categorical variables are presented using descriptive statistics, including frequencies and percentages. Continuous variables are reported as means with standard deviations or medians with ranges, as appropriate. The Wilcoxon signed-rank test was used to assess changes in quality-of-life parameters before and after surgery, with a p value < 0.05 considered statistically significant.

Subgroup analyses were conducted to compare outcomes across age categories (pediatric, adolescent, and adult), between sexes, and between patients with cosmetic versus physiological surgical indications. The Mann–Whitney U test was used for comparisons between independent groups, and Spearman’s rank correlation was used to assess associations between preoperative factors (e.g., chest pain, age at surgery) and postoperative satisfaction.

Patients were secondarily grouped based on the number of bars utilized (one vs. two), and descriptive complication rates were compared across these subgroups. Additionally, response distributions for SSQ questions 14 and 16 were analyzed to quantify overall and decision satisfaction.

All statistical analyses were performed using SPSS software version 25.0 (IBM Corp., Armonk, NY, USA). Due to limited sample size, subgroup comparisons were presented descriptively without inferential testing beyond primary variables.

## Results

The results presented in Table 2 provide a comprehensive descriptive analysis of the study population (*n* = 40) who underwent the Nuss procedure for pectus excavatum correction, with a mean follow-up period of 108.53 ± 54.74 months, as shown in Table 2. The demographic data revealed that the study cohort predominantly consisted of male patients (77.5%), with a mean age at operation of 16.32 ± 4.03 years, reflecting the typical demographic pattern of pectus excavatum patients. Preoperative symptomatology was significant, with dyspnea being the most common symptom (75.0%), followed by palpitation (57.5%), exercise intolerance (55.0%), and chest pain (42.5%). The primary indication for surgical intervention was physiological needs (85.0%), whereas cosmetic needs were the main indication in only 15.0% of cases. The mean interval between the operation and assessment was substantial at 108.53 ± 54.74 months (approximately 9 years), providing a long-term perspective on surgical outcomes. The majority of patients required a single bar for correction (mean bar number: 1.05 ± 0.22). Postoperative complications are relatively uncommon, with both pneumothorax and chronic pain occurring in only 7.5% of patients.

**Table 2:** Descriptive analysis of the study population (*n* = 40)

Variable	Result
Age At Operation (year)	16.32 ± 4.03
Gender Female Male	9 (22.5%) 31 (77.5%)
Dyspnea	30 (75.0%)
Chest Pain	17 (42.5%)
Exercise Intolerance	22 (55.0%)
Palpitation	23 (57.5%)
Indication Physiological Needs	34 (85.0%)
Indication Cosmetic Needs	6 (15.0%)
Complication Pneumothorax	3 (7.5%)
Complication Chronic Pain	3 (7.5%)
Interval Between Operation and Assessment (months)	108.53 ± 54.74
Used Bar Number	1.05 ± 0.22
SSQ-Physical Functioning Score	8.60 ± 1.56
SSQ-Social Functioning Score	10.88 ± 2.22
SSQ-Cosmetic Satisfaction Score	7.97 ± 1.57
SSQ-Cosmetic Outcome Score	4.28 ± 0.74
SSQ-Self Esteem Score	2.35 ± 2.16
SSQ-Pain Discomfort Score	8.45 ± 2.16
SSQ-Physical Awareness Score	2.73 ± 1.32
SSQ-Overall Satisfaction Score	4.38 ± 0.83
SSQ-Decision Satisfaction Score	9.25 ± 2.38
SSQ-Total Score	50.27 ± 7.78

Continuous variables are presented as mean ± standard deviation (SD); categorical variables as number (percentage)

The single-step questionnaire (SSQ) scores generally reflect positive outcomes across multiple domains. Social functioning (10.88 ± 2.22) and physical functioning (8.60 ± 1.56) demonstrated favorable results. The cosmetic satisfaction score (7.97 ± 1.57) and cosmetic outcome score (4.28 ± 0.74) indicated high patient satisfaction with the aesthetic results. The self-esteem score (2.35 ± 2.16) and physical awareness score (2.73 ± 1.32) were relatively low, which may reflect ongoing body image concerns despite surgical correction. The pain/discomfort score (8.45 ± 2.16) suggested good long-term pain resolution. The total SSQ score (50.27 ± 7.78) represents the aggregate patient-reported outcomes across the multiple quality-of-life dimensions. Overall satisfaction was notably high (4.38 ± 0.83), and decision satisfaction was near the maximum (9.25 ± 2.38).

### Analysis of the impact of clinical variables

Patients were stratified into three age groups: children (≤ 12 years), adolescents (13–17 years), and adults (≥ 18 years). The demographic and clinical characteristics of the study population stratified by age group are presented in Table 3. There were significant differences in age distribution among the three groups, while the sex distribution remained similar across all groups. Dyspnea was the most common preoperative symptom across all age groups, followed by palpitation, exercise intolerance, and chest pain.

**Table 3:** Patient characteristics stratified by age group

Variable	Children (*n* = 10)	Adolescents (*n* = 18)	Adults (*n* = 12)	*p* value
Age (years)	11.0 (9–12)	16.0 (13–17)	21.5 (18–26)	< 0.001
Gender (Male)	8 (80.0%)	14 (77.8%)	9 (75.0%)	0.953
Dyspnea	7 (70.0%)	14 (77.8%)	9 (75.0%)	0.898
Chest Pain	3 (30.0%)	7 (38.9%)	7 (58.3%)	0.374
Exercise Intolerance	5 (50.0%)	9 (50.0%)	8 (66.7%)	0.617
Palpitation	4 (40.0%)	10 (55.6%)	9 (75.0%)	0.238
Physiological Indication	8 (80.0%)	15 (83.3%)	11 (91.7%)	0.717
Cosmetic Indication	2 (20.0%)	3 (16.7%)	1 (8.3%)	0.717
Interval Between Operation and Assessment (months)	92.5 (62–195)	96.5 (60–240)	98.0 (60–238)	0.944
Bars Utilized	1.0 (1–1)	1.0 (1–2)	1.0 (1–2)	0.667

Continuous variables are presented as mean ± SD or median (range), depending on data distribution. Categorical variables are shown as number (percentage)

Analysis of the SSQ scores across age groups revealed significant differences in multiple domains, as shown in Table 4. The pediatric group had significantly higher scores in physical functioning, pain/discomfort, and total SSQ score compared to other age groups (*p* < 0.05). Postoperative complications, including pneumothorax and chronic pain, were observed exclusively in the adolescent and adult groups. No complications occurred in pediatric patients. Chronic pain was reported only in the adult cohort (25.0%) (Table 5). Due to limited subgroup sizes, no statistical testing was performed. The sex-based comparison of the SSQ scores is presented in Table 6. There was no statistically significant difference between male and female patients in self-esteem and social functioning scores (*p* > 0.05). Correlation analysis between age at operation and SSQ score revealed significant negative associations, as depicted in Table 7. Increasing age at operation was significantly correlated with lower scores in the physical functioning, pain/discomfort, and total SSQ domains. Children had significantly higher SSQ scores in physical functioning, pain/discomfort, and total score compared to adolescents and adults (*p* < 0.05). Analysis of the relationship between preoperative symptoms and long-term satisfaction revealed that preoperative chest pain was significantly associated with lower total SSQ scores, as shown in Table 8. A comparison of outcomes based on the primary indication for surgery is presented in Table 9. Patients whose primary indication was cosmetic had significantly higher self-esteem and total SSQ scores compared to those with physiological indications (*p* < 0.05).

**Table 4:** Comparison of SSQ scores across age groups

SSQ Domain	Children (*n* = 10)	Adolescents (*n* = 18)	Adults (*n* = 12)	*p* value
Physical Functioning	10.0 (8–10)	9.0 (6–10)	8.0 (5–10)	0.018*
Social Functioning	11.5 (8–15)	11.0 (7–15)	10.5 (7–14)	0.654
Cosmetic Satisfaction	9.0 (6–10)	8.0 (5–10)	7.5 (5–10)	0.391
Cosmetic Outcome	5.0 (3–5)	4.5 (3–5)	4.0 (3–5)	0.444
Self-Esteem	3.5 (0–6)	2.0 (0–6)	1.5 (0–5)	0.158
Pain/Discomfort	10.5 (8–12)	8.5 (5–12)	7.0 (4–11)	0.003**
Physical Awareness	3.0 (1–5)	3.0 (1–5)	2.5 (1–4)	0.774
Overall Satisfaction	5.0 (3–5)	5.0 (2–5)	4.0 (2–5)	0.228
Decision Satisfaction	10.0 (10–10)	10.0 (0–10)	10.0 (0–10)	0.417
Total SSQ Score	56.5 (48–61)	51.0 (32–62)	45.5 (33–59)	0.012*

Continuous variables are presented as median (range)

* *p* < 0.05, ** *p* < 0.01, *** *p* < 0.001

**Table 5:** Postoperative complication rates stratified by age group

Age Group	*n*	Pneumothorax (*n*, %)	Chronic Pain (*n*, %)	Total Complications (*n*, %)
Children (≤ 12)	10	0 (0.0%)	0 (0.0%)	0 (0.0%)
Adolescents	18	2 (11.1%)	0 (0.0%)	2 (11.1%)
Adults (≥ 18)	12	1 (8.3%)	3 (25.0%)	4 (33.3%)

Due to small sample sizes in each subgroup, formal statistical tests were not performed. However, complication rates showed an increasing trend with age, particularly with chronic pain observed exclusively in the adult group

**Table 6:** Comparison of SSQ scores by gender

SSQ Domain	Male (*n* = 31)	Female (*n* = 9)	*p* value
Physical Functioning	9.0 (5–10)	9.0 (6–10)	0.723
Social Functioning	11.0 (7–15)	12.0 (8–14)	0.231
Cosmetic Satisfaction	8.0 (5–10)	9.0 (6–10)	0.324
Cosmetic Outcome	4.0 (3–5)	5.0 (3–5)	0.453
Self-Esteem	2.0 (0–6)	3.0 (0–6)	0.296
Pain/Discomfort	9.0 (4–12)	7.0 (5–12)	0.167
Physical Awareness	3.0 (1–5)	3.0 (1–5)	0.893
Overall Satisfaction	5.0 (2–5)	5.0 (3–5)	0.473
Decision Satisfaction	10.0 (0–10)	10.0 (5–10)	0.782
Total SSQ Score	51.0 (32–62)	53.0 (42–59)	0.510

**Table 7:** Correlation between age at operation and SSQ score

SSQ Domain	Correlation Coefficient (*r*)	*p* value
Physical Functioning	−0.321	0.043*
Social Functioning	−0.156	0.336
Cosmetic Satisfaction	−0.283	0.077
Cosmetic Outcome	−0.225	0.163
Self-Esteem	−0.302	0.058
Pain/Discomfort	−0.342	0.031*
Physical Awareness	−0.118	0.468
Overall Satisfaction	−0.178	0.271
Decision Satisfaction	−0.304	0.056
Total SSQ Score	−0.355	0.025*

* *p* < 0.05, ** *p* < 0.01, *** *p* < 0.001

**Table 8:** Correlations between preoperative symptoms and SSQ scores

Symptom	Correlation with Total SSQ Score (*r*)	*p* value
Dyspnea	−0.187	0.248
Chest Pain	−0.322	0.043*
Exercise Intolerance	−0.276	0.085
Palpitation	−0.212	0.190

* *p* < 0.05, ** *p* < 0.01, *** *p* < 0.001

**Table 9:** Comparison of SSQ scores by primary indication

SSQ Domain	Physiological (*n* = 34)	Cosmetic (*n* = 6)	*p* value
Physical Functioning	9.0 (5–10)	9.0 (7–10)	0.485
Social Functioning	11.0 (7–15)	12.5 (10–14)	0.173
Cosmetic Satisfaction	8.0 (5–10)	9.0 (7–10)	0.149
Cosmetic Outcome	4.0 (3–5)	5.0 (4–5)	0.163
Self-Esteem	2.0 (0–6)	4.0 (2–6)	0.041*
Pain/Discomfort	8.5 (4–12)	9.5 (6–12)	0.498
Physical Awareness	3.0 (1–5)	3.0 (2–5)	0.591
Overall Satisfaction	4.5 (2–5)	5.0 (4–5)	0.136
Decision Satisfaction	10.0 (0–10)	10.0 (10–10)	0.361
Total SSQ Score	49.5 (32–62)	56.5 (51–61)	0.043*

* *p* < 0.05, ** *p* < 0.01, *** *p* < 0.001

To identify independent predictors of patient satisfaction, multiple regression analysis was performed with the total SSQ score as the dependent variable. As shown in Table 10, age at operation, preoperative chest pain, and postoperative chronic pain emerged as significant independent predictors of lower total SSQ scores. The model explained 38.7% of the variance in satisfaction outcomes, providing a moderately robust framework for identifying patients at risk for suboptimal long-term satisfaction. Among the 40 patients, 36 (90%) received a single bar and 4 (10%) received two bars. Among the 40 patients, 36 (90%) received a single bar and 4 (10%) received two bars. The subgroup with two bars had higher observed rates of complications, including pneumothorax and chronic pain. Due to the small sample size, no statistical comparison was performed (Table 11). The distribution of responses to SSQ questions 14 (overall satisfaction) and 16 (decision satisfaction) are shown in Table 12. The majority of patients reported high levels of satisfaction, with 80% of participants selecting either “extremely satisfied” or “very satisfied.” Regarding decision satisfaction, 75% of patients stated that they would choose to undergo the operation again.

**Table 10:** Multiple regression analysis of factors influencing total SSQ scores

Variable	Standardized Beta	*p* value
Age at Operation	−0.326	0.038*
Gender (Female)	0.112	0.462
Preoperative Chest Pain	−0.315	0.042*
Preoperative Exercise Intolerance	−0.257	0.087
Complication (Chronic Pain)	−0.355	0.022*
Interval Since Operation	0.072	0.635
Model R² = 0.387, Adjusted R² = 0.309, *p* = 0.003**		

* *p* < 0.05, ** *p* < 0.01, *** *p* < 0.001

**Table 11:** Relationship between number of bars used and postoperative complications

Bars Utilized	Number of Patients	Pneumothorax (*n*, %)	Chronic Pain (*n*, %)	Total Complication Rate (%)
1 bar	36 (90.0%)	1 (2.8%)	1 (2.8%)	2 (5.6%)
2 bars	4 (10.0%)	2 (50.0%)	2 (50.0%)	3 (75.0%)

**Table 12:** Distribution of responses to SSQ questions 14 (Overall Satisfaction) and 16 (Decision Satisfaction)

SSQ Question	Response Option	*n* (%)
Q14: Overall Satisfaction	Extremely satisfied (5)	18 (45.0%)
	Very satisfied (4)	14 (35.0%)
	Satisfied (3)	6 (15.0%)
	Dissatisfied (2)	2 (5.0%)
	Very dissatisfied (1)	0 (0.0%)
Q16: Decision Satisfaction	Yes (10)	30 (75.0%)
	Unsure (5)	8 (20.0%)
	No (0)	2 (5.0%)

## Discussion

Our retrospective analysis of 40 patients who underwent the Nuss procedure for pectus excavatum correction revealed favorable long-term outcomes, with high patient satisfaction persisting at a median follow-up of 96.5 months postoperation. The study population predominantly consisted of male patients, reflecting the established epidemiological profile of pectus excavatum. When stratified by age groups, the demographic and clinical characteristics were similar across the pediatric, adolescent, and adult cohorts, with dyspnea emerging as the most common preoperative symptom in all groups. While children demonstrated statistically higher SSQ scores in certain domains, the absolute differences were relatively small. Despite statistical significance, these differences may not reflect clinically meaningful distinctions. Gender-based comparisons revealed no significant differences between male and female patients across any of the SSQ domains, although female patients had marginally higher scores for self-esteem and social functioning. The negative correlation observed between age at operation and multiple SSQ domains, including total SSQ scores, further supported the age-dependent pattern of outcomes. Analysis of preoperative symptoms revealed that chest pain was associated with lower overall satisfaction, suggesting that this particular symptom might indicate a more complex underlying pathophysiology not fully addressed by structural correction. We observed that primary surgical indications influenced satisfaction outcomes, with patients whose primary indication was cosmetic demonstrating higher self-esteem scores and total SSQ scores than those whose primary indication was physiological. Patients who developed chronic pain as a complication presented markedly lower scores across multiple domains, particularly for pain/discomfort, overall satisfaction, and decision satisfaction. However, due to the small number of affected cases (*n* = 3) and lack of stratified SSQ data, we were unable to conduct a formal statistical comparison between patients with and without chronic pain. This represents a limitation of the current study and suggests the need for more detailed, prospective assessments of complication-linked satisfaction outcomes. Although chronic pain was more frequently reported in the adult group, this may reflect a combination of factors, including surgical technique, implant configuration, and patient-specific anatomical features. Therefore, the observed association between age and pain should be interpreted with caution.

Finally, our multiple regression analysis identified three independent predictors of lower satisfaction: advanced age at operation, preoperative chest pain, and postoperative chronic pain. These findings collectively suggest that earlier intervention might optimize long-term outcomes and that effective management of both preoperative and postoperative pain is crucial for maximizing patient satisfaction following the Nuss procedure.

The age-dependent outcomes and satisfaction metrics observed in our study align with several recent publications. Wang et al. demonstrated in their propensity score-matched study that early surgical intervention (4–6 years) compared with adolescent intervention (12–14 years) did not increase recurrence rates or reduce long-term satisfaction but significantly lowered postoperative analgesic requirements and hospital stay duration [13]. Our findings extend this observation by demonstrating a more comprehensive age-dependent gradient in satisfaction, with pediatric patients showing superior outcomes across multiple quality-of-life domains compared with both adolescents and adults. This pattern suggests a potential benefit to earlier intervention when it is clinically appropriate. Similarly, a comprehensive analysis by Rshaidat et al. using the TriNetX research network revealed significant differences in complication rates between adult and pediatric populations, with adults experiencing higher rates of hemorrhagic complications (3% vs. 0.86%) and acute postoperative pain (55% vs. 39.1%) [14]. This finding corresponds with our observation of higher chronic pain incidence in the adult cohort, although our smaller sample size resulted in this trend not reaching statistical significance. Furthermore, while statistical comparison was not feasible due to small sample sizes, complication rates appeared to increase with age. The absence of complications in pediatric patients and the presence of chronic pain exclusively in the adult group underscore the potential benefits of early intervention.

The critical impact of postoperative pain management on long-term outcomes is highlighted by our finding that chronic pain significantly impaired satisfaction across multiple domains. This observation aligns with the comprehensive review by Chiu et al., who emphasized multimodal approaches, including intercostal nerve cryoablation (INC), regional nerve blocks, and enhanced recovery after surgery (ERAS) protocols. Their review highlighted how these advanced techniques reduce opioid consumption, shorten hospital stays, and improve postoperative recovery. Chiu et al. noted that INC provides sustained analgesia while minimizing opioid-related complications, although its delayed onset necessitates complementary early postoperative pain control measures [15]. The application of these techniques may be particularly beneficial for adult patients, who demonstrated greater vulnerability to chronic pain in both our study and the analysis by Rshaidat et al. Regarding sex-specific outcomes, our study revealed no significant differences between male and female patients, although female patients showed marginally greater improvements in self-esteem. This contrasts somewhat with Rshaidat et al., who reported higher overall complication rates in female patients (28.48% vs. 21.7%), including increased respiratory complications (6% vs. 2.7%), chronic pain (5.2% vs. 2%), and hemorrhagic complications (6% vs. 0.97%). Their study also noted that female patients tended to present for surgical repair at an older age than did male patients [14]. This discrepancy with our findings may be attributed to our relatively small female cohort (*n* = 9), limiting the statistical power to detect significant sex-based differences. The findings of Jaroszewski et al. in their development of the PCAPES questionnaire provide additional context, as they reported that female and older patients presented more severe cardiac symptoms. An observational study of 432 patients revealed a high prevalence of various symptoms, including headaches (74%), positional dizziness (67%), exercise limitations (> 70%), restricted breathing (> 80%), and psychosocial impacts, with 80% of patients being bothered by chest appearance [16]. These sex-specific manifestations may influence surgical outcomes and warrant further investigation in larger, sex-balanced cohorts.

Our finding that cosmetic indications were linked to greater satisfaction than physiological indications aligns with the findings of Higaze et al., who reported that MIRPE significantly improved both physical and mental well-being, including physical function, pain reduction, and psychosocial factors [17]. The cosmetic improvements achieved through surgery appear to provide substantial psychological benefits that meet or exceed patient expectations. However, the lower self-esteem scores we observed in our overall cohort suggest that psychological healing may progress more slowly than physical recovery does, emphasizing the need for comprehensive postoperative care addressing both physical and emotional aspects. The technical aspects of the Nuss procedure substantially influence patient outcomes, as demonstrated by Skrzypczak et al., who reported that while patients with one or two bars achieved similar corrective outcomes, those with two bars experienced significantly higher rates of complications, including pneumothorax, pleural effusion, and bar displacement [18]. In our cohort, the majority of patients received a single bar, which may have contributed to the relatively low overall complication rate. These findings highlight the need for careful patient selection and surgical planning to balance optimal correction with minimizing postoperative risk. The importance of technical expertise is further underscored by Notrica, who identified 20 common error traps in patient selection, intraoperative technique, and postoperative care. Key pitfalls included inappropriate patient selection, failure to anticipate anatomical variations, inadequate bar fixation, and mismanagement of postoperative pain. Notably, thoracic epidural analgesia, once widely used, provides no significant advantage over alternative pain control strategies such as intercostal nerve cryoablation [19].

Although our study did not specifically examine this relationship, the low complication rates in our cohort likely reflect our institution’s expertise in performing these procedures. Surgical technique selection significantly impacts outcomes, as demonstrated by Narkhojayev et al. in their study comparing three thoracoplasty methods in 183 pediatric patients. Their research revealed that while the Nuss procedure offered the shortest operating time, it was associated with risks of atelectasis and pneumothorax. In contrast, modified thoracoplasty with intercostal blockade results in the shortest period of postoperative pain [20]. These findings confirm that the Nuss procedure remains safe and effective when performed with the proper technique and careful patient selection. The durability of satisfaction after the Nuss procedure observed in our study mirrors the findings of Sacco Casamassima et al., who evaluated long-term outcomes in 98 adult patients (median age: 30.9 years) who underwent a modified Nuss procedure between 1998 and 2011. They reported that 89.7% of patients were satisfied with their chest appearance, and 84.6% noted improvement in social interaction. Similar to our findings, where 80% of patients reported being either extremely or very satisfied (Q14) and 75% stated they would have the operation again (Q16), 82% of patients in their cohort reported high satisfaction and 79.5% indicated willingness to undergo the procedure again. They concluded that although Nuss repair may be more challenging in adults in terms of postoperative pain control, the procedure ensures satisfactory reconfiguration of the chest wall comparable to that achieved in pediatric patients [21]. Our comparable findings in pediatric and adolescent populations extend these observations across a broader age spectrum and suggest that despite age-dependent variations in outcome magnitude, the Nuss procedure yields generally favorable long-term satisfaction across all age groups.

### Strengths and limitations

This study’s primary strengths include its extended follow-up period (median 96.5 months), the inclusion of age-stratified analyses across pediatric, adolescent, and adult groups, and the use of a validated patient-reported outcome measure (SSQ). Additionally, the identification of independent predictors of satisfaction through multivariate analysis enhances its clinical relevance.

However, limitations include the small sample size and uneven age group distribution, which may reduce the statistical power of subgroup comparisons. The retrospective, single-center design introduces risks of selection and recall bias and may limit generalizability. Furthermore, reliance on subjective outcome measures and the underrepresentation of female patients restricts interpretation of sex-specific results.

## Conclusions

Our long-term evaluation of patients who underwent the Nuss procedure for pectus excavatum demonstrated durable satisfaction across multiple quality-of-life domains approximately nine years postoperation, with particularly favorable outcomes in pediatric patients. The age-dependent pattern of outcomes, with younger patients exhibiting better satisfaction and lower complication rates, supports the consideration of earlier intervention when clinically appropriate. Preoperative chest pain and postoperative chronic pain emerged as significant negative predictors of long-term satisfaction, highlighting the importance of comprehensive pain management strategies. Patients who underwent correction for primarily cosmetic indications reported greater satisfaction than those with physiological indications did, suggesting that alignment between expectations and outcomes influences perceived benefit. These findings contribute to evidence-based decision-making regarding the optimal timing of intervention, patient selection, and perioperative management for pectus excavatum correction and support the Nuss procedure as an effective approach for appropriately selected patients.

## Data Availability

No datasets were generated or analysed during the current study.
